# A Streamlined Approach to Rapidly Detect SARS-CoV-2 Infection Avoiding RNA Extraction: Workflow Validation

**DOI:** 10.1155/2020/8869424

**Published:** 2020-12-09

**Authors:** Catia Mio, Adriana Cifù, Stefania Marzinotto, Natascha Bergamin, Chiara Caldana, Silvia Cattarossi, Sara Cmet, Annarosa Cussigh, Romina Martinella, Jessica Zucco, Roberto Verardo, Claudio Schneider, Barbara Marcon, Stefania Zampieri, Corrado Pipan, Francesco Curcio

**Affiliations:** ^1^Department of Medicine (DAME), University of Udine, Udine, Italy; ^2^Department of Laboratory Medicine, University Teaching Hospital of Udine, Udine, Italy; ^3^Consorzio Interuniversitario Biotecnologie (CIB), Trieste, Italy

## Abstract

Severe acute respiratory syndrome coronavirus 2 (SARS-CoV-2) infection has rapidly spread worldwide from the beginning of 2020. The presence of viral RNA in samples by nucleic acid (NA) molecular analysis is the only method available to diagnose COVID-19 disease and to assess patients' viral load. Since the demand for laboratory reagents has increased, there has been a worldwide shortage of RNA extraction kits. We, therefore, developed a fast and cost-effective viral genome isolation method that, combined with quantitative RT-PCR assay, detects SARS-CoV-2 RNA in patient samples. The method relies on the addition of Proteinase K followed by a controlled heat-shock incubation and, then, *E gene* evaluation by RT-qPCR. It was validated for sensitivity, specificity, linearity, reproducibility, and precision. It detects as low as 10 viral copies/sample, is rapid, and has been characterized in 60 COVID-19-infected patients. Compared to automated extraction methods, our pretreatment guarantees the same positivity rate with the advantage of shortening the time of the analysis and reducing its cost. This is a rapid workflow meant to aid the healthcare system in the rapid identification of infected patients, such as during a pathogen-related outbreak. For its intrinsic characteristics, this workflow is suitable for large-scale screenings.

## 1. Introduction

In late December 2019, some patients affected by viral pneumonia were found to be epidemiologically associated with the Huanan seafood market in Wuhan, China [1, 2]. Rapidly, through next-generation sequencing (NGS), a novel human-infecting coronavirus named severe acute respiratory syndrome coronavirus 2 (SARS-CoV-2) was isolated, and by the beginning of 2020, the World Health Organization (WHO) declared the pandemic outbreak [3, 4]. It is a single-stranded RNA virus belonging to the *Betacoronavirus* genus. Multiple sequence alignments revealed that SARS-CoV-2 is closely related to bat-derived SARS-like coronaviruses (89% similarity), but when compared with severe acute respiratory syndrome coronavirus (SARS-CoV) and middle east respiratory syndrome (MERS-CoV), SARS-CoV-2 showed less genetic similarity (79% and 50%, respectively) [5]. The associated disease, named COVID-19, is characterized by fever, fatigue, dry cough, pharyngodynia, shortness of breath, headache, chest tightness, chest pain, and myalgia. Some of SARS-CoV-2 patients have rhinorrhea, nausea, vomiting, and diarrhea [3]. In some patients, symptomatology rapidly exacerbates leading to acute respiratory distress syndrome (ARDS) and multiple organ dysfunction syndromes (MODS) [6]. A variable proportion of infected subjects, estimated about 20-25%, do not display any symptoms, making it difficult to limit the infection. Indeed, these patients can spread the virus and may represent a population that can be easily neglected in epidemic prevention. Indeed, public health authorities need to rapidly implement quick and sensitive diagnostic tools for patients' management.

Viral RNA detection is the main, fastest, and most sensitive test for the diagnosis of SARS-CoV-2 infection [7]. An approach using nucleic acid extraction in combination with quantitative PCR (qPCR) was rapidly developed with successful detection of affected patients. Several NA-automated analytical molecular systems are available, which, although having the advantage of obtaining a pure product of the highest quality, are expensive and greatly lengthen the analysis procedures [8]. With the exponential growth of infections, the demand for laboratory reagents greatly increased. In particular, a dramatic worldwide shortage in RNA extraction kits was experienced [9], greatly impairing our ability to limit the spreading of the pandemic with tragic consequences. To solve the above-mentioned issue, we developed a procedure for the treatment of nasopharyngeal swab, collected from patients with suspected SARS-CoV-2 infection (*N* = 500), which eliminates the need for the RNA extraction step. The aim of the current study was, then, to validate an in-house method for isolating viral RNA from nasopharyngeal swabs to be tested by quantitative reverse transcriptase quantitative PCR (RT-qPCR).

## 2. Material and Methods

### 2.1. Patient Samples and RNA Isolation

For SARS-CoV-2 RNA detection, samples from 30 positive and 30 negative subjects were collected with UTM® tubes (COPAN Diagnostics Inc.). Ethical approval was obtained from the Medical Research Ethics Committee of the Region Friuli Venezia Giulia, Italy (CEUR-2020-Os-033). In a 96-well plate, 10 *μ*L of a 30 mg/mL 5 (Merck KGaA) solution in Hanks' Balanced Salt Solution (HBSS) w/calcium and magnesium (Merck) was added to 100 *μ*L of UTM® tube-derived medium. The plate was heated for 15 min at 55°C, denatured for 5 min at 98°C, and then placed for 2 min at 4°C.

### 2.2. Gold Standard Extraction Method

As a gold standard method for viral NA extraction, the ELITe InGenius® SP200 System (ELITech Group SAS) was used, following the manufacturer's instructions: 200 *μ*L of medium from 3 mL of UTM® tube was used as the template, and samples were eluted in 100 *μ*L elution buffer.

### 2.3. Quantitative Reverse Transcription Polymerase Chain Reaction (RT-qPCR)

To detect viral RNA, LightMix® Modular SARS and Wuhan CoV E-gene (Roche) were used. Briefly, 4 *μ*L Roche Master, 0.5 *μ*L Reagent mix (containing primers and probes for the *envelope* (E) *gene* according to the German Consiliary Laboratory for Coronaviruses (Charité, Berlin)) [10], 0.1 *μ*L RT enzyme, 5 *μ*L sample/positive control, and nuclease-free water were mixed to a total volume of 15 *μ*L. The human *RNase P* gene primer and probe set were used as an internal positive control to monitor sample quality, and RNA isolation. RT-qPCR was performed by the LightCycler® 480 II Instrument (Roche), and absolute quantification was assessed by the LightCycler® 480 II System (Roche).

### 2.4. Validation Parameters and Statistical Analysis

#### 2.4.1. Limit of Detection (LoD)

The LoD was assessed by 5 serial dilutions (5-fold each) of viral RNA isolated from a positive patient with the in-house method and assessed by RT-qPCR. Each standard point was analyzed in 10 replicates. In our case, LoD represents the lowest amount of copies of the *E gene* in a given sample that can be clearly distinguished from the background with 95% probability, ensuring a false positive rate ≤ 5%. The Grubb's test was used to identify the outliers, and 46 data points were used for the analysis. The distribution of data was checked with the Shapiro-Wilk test.

#### 2.4.2. Accuracy

To assess sensitivity and specificity of the in-house method, a total of 60 samples, divided in 30 SARS-CoV-2-positive and 30-negative samples, were used.

#### 2.4.3. Agreement

Bland-Altman analysis was used to evaluate the agreement between our in-house method and the gold standard (Elite InGenius SP200 System-mediated automated RNA extraction) [11]. Moreover, a comparison between extraction methods was made using the Spearman rank correlation coefficient analysis. The level of agreement was measured using Cohen's kappa method.

#### 2.4.4. Linearity

The linear range of the in-house method was established with 7 serial dilutions of viral RNA assessed by RT-qPCR. Each dilution was tested 4 times. The relationship between the observed values and the one obtained with the gold standard was examined with the linear regression. The efficiency was calculated based on the slope of the dilution series.

#### 2.4.5. Precision

The precision was estimated by performing the RT-qPCR of the same sample under specific conditions. Repeatability was assessed by testing a positive sample 3 times on the same day at 3 different concentrations. Reproducibility (intermediate precision) was evaluated by analyzing the same sample 3 times each day for 3 consecutive days at different concentrations. The Shapiro-Wilk test was used to check the distribution of each series. Levene's test was used to assess the equality of variances in repeatability test and intermediate precision was assessed by one-way ANOVA according to ISO 5725 guidelines.

All statistical analyses were performed with GraphPad Prism 6.0. A *p* value ≤ 0.05 was considered statistically significant (^∗^*p* < 0.05, ^∗∗^*p* < 0.01, ^∗∗∗^*p* < 0.001, and ^∗∗∗∗^*p* < 0.0001).

## 3. Results

**Figure 1:**
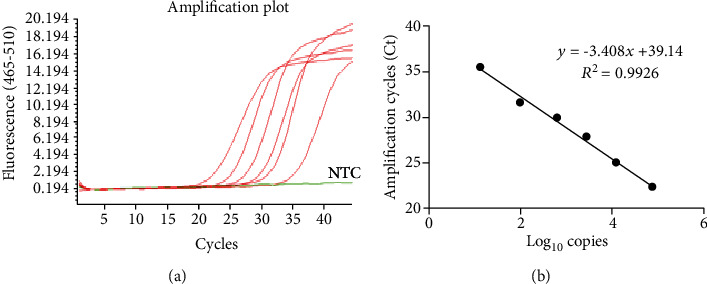
Standard curve made by diluting the SARS-CoV-2-positive control. (a) Shows data from 7 serial dilutions (100%, 20%, 10%, 2%, 1%, 0.5%, and 0.1%) of the SARS-CoV-2-positive control (PC) supplied with the LightMix® Modular kit evaluated with RT-qPCR. The 0.1% dilution showed no amplification curve as NTC. Panel B shows the standard curve made by the 7 dilutions of the PC. NTC: no template control.

**Figure 2:**
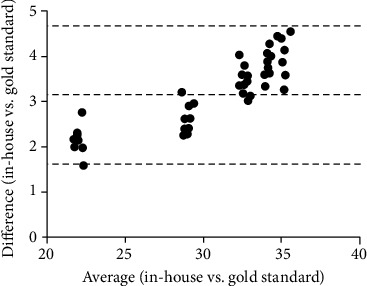
Agreement between the in-house method and the gold standard. Bland-Altman's diagram shows the difference between the two methods on the *y*-axis and their average on the *x*-axis. The bias between the measurements was 3.16 (CI95% 1.62-4.69) with 95% agreement. The dotted lines represent the bias and the upper and lower limits of 95% agreement.

The result of LoD assessment is shown in Figure 1(a). The LoD was fixed at 36 amplification cycles (Ct), corresponding to about 10 copies when this value is interpolated on a standard curve obtained by diluting a positive control supplied with the LightMix® Modular kit (Figure 1(b)). Therefore, we set a threshold of 36 Ct to discriminate between positive and negative samples. The sensitivity and specificity for the in-house method were calculated analyzing 60 samples (*N* = 30 positive and *N* = 30 negative using the gold standard method).  Table 1 summarizes the data obtained with RT-qPCR from 30 positive swabs. All negative (*N* = 30) samples showed no amplification profile. RT-qPCR was performed, and after filtering data with the aforementioned threshold value, 90% sensibility and 100% specificity were found (Table 2). The Bland-Altman test showed that the bias between the measurements was 3.16 (CI95% 1.62-4.69) with an agreement of 95% (Figure 2). The Spearman's rho test showed a high correlation between the two methods (*r* = 0.9116, *p* < 0.0001). To further assess agreement, Cohen's kappa was calculated. This is a statistical coefficient representing the degree of accuracy, ranging from 0.01 to 1.00. Referring to data in Table 1, Cohen's kappa was 0.90. Since the value is in the highest part of the range (0.81-1.00), it ensures an almost perfect degree of agreement.

The extent of linearity of our method was determined by serial dilutions of a SARS-CoV-2-positive specimen (4 replicates per dilution, 7 dilutions). The results of linear regression are summarized in Table 3, including the parameters of the fitted models.

Repeatability was evaluated by testing 3 dilutions of a SARS-CoV-2-positive sample 3 times daily. The *p* value calculated with Levene's test was *p* = 0.17 indicating that there was no significant difference between the measurements. Intermediate precision was evaluated with the same set of dilutions reproduced for 3 consecutive days. The results are shown in Table 4.

## 4. Conclusion

Following the declaration of pandemic in early 2020, national and local public health laboratories have addressed the crucial problem of detecting the newly isolated pathogen [12], in order to rapidly identify infected individuals and implement containment strategies to prevent its spread. There was soon a shortage of RNA extraction kits, which is the first step in performing the molecular test for the detection of SARS-CoV-2 [9]. Due to this deficiency and the huge amount of samples to be processed daily, RNA extraction from nasopharyngeal swabs of COVID-19 patients has become a bottleneck in diagnostic procedures. In many cases, this deficiency has led to limiting the test to patients with (often nonspecific) symptoms. As a result, many asymptomatic or paucisymptomatic individuals [6] were not tested, thus becoming a major vehicle for the spread of the infection, first in China [13] and then in the rest of the world.

Here, we describe a rapid and cost-effective method that can be applied to analyze swab-derived viral RNA. The strength of this method is its ability to detect as low as 10 copies of the SARS-CoV-2 NA with high sensitivity and specificity (90% and 100%, respectively). An obvious advantage compared to the gold standard (i.e., the automated extraction of RNA using the EliTe inGenius SP200 System followed by RT-qPCR) is certainly that this in-house method is faster, allowing to process 96 samples in about 20 minutes halving the processing time of the samples. Furthermore, by avoiding RNA extraction, the cost per sample is drastically reduced. These features make it useful for rapid sample screening, as in a pandemic outbreak. To validate this method, we have used primers and assays with probes targeted to the *E gene*, indicated as the most sensitive combination of Roche's modular LightMix® kit. This is a key step as identifying infected subjects immediately triggers containment strategies to reduce the chance of infection. Among the most important performance parameters for a diagnostic procedure are those related to the minimum amount of target that can be detected [14]. Our in-house method possesses an LoD of 10 copies of RNA, with an agreement with the gold standard method of 95%. In addition, since a key feature in diagnostic procedures is precision, we have evaluated repeatability and reproducibility by testing several dilutions of a positive samples every day for three days in a row. The coefficient of variation (CV) was less than 25% both inter- and intraday indicating that there was no significant difference in results between the results (*p* = 0.17).

In summary, we have developed a SARS-CoV-2 extraction-free isolation method suitable for RT-qPCR detection with the *E gene* assay. This is a rapid workflow that can help the healthcare system in the prompt identification of infected patients, as is necessary during a pathogen-related outbreak.

## Figures and Tables

**Table 1:** Amplification data of 30 positive samples extracted with the two methods.

Sample ID	Home-made RNA isolation (Ct)	Automated RNA extraction (Ct)
SARS-CoV-2 #1	21.08	20.10
SARS-CoV-2 #2	22.56	20.84
SARS-CoV-2 #3	24.52	21.97
SARS-CoV-2 #4	35.60	34.13
SARS-CoV-2 #5	36.20	34.03
SARS-CoV-2 #6	36.10	35.61
SARS-CoV-2 #7	35.60	32.70
SARS-CoV-2 #8	34.60	32.64
SARS-CoV-2 #9	35.20	33.30
SARS-CoV-2 #10	35.60	35.06
SARS-CoV-2 #11	34.70	32.32
SARS-CoV-2 #12	34.50	32.88
SARS-CoV-2 #13	35.50	32.42
SARS-CoV-2 #14	35.50	33.18
SARS-CoV-2 #15	32.80	29.70
SARS-CoV-2 #16	29.30	27.98
SARS-CoV-2 #17	33.70	28.29
SARS-CoV-2 #18	31.15	29.83
SARS-CoV-2 #19	35.90	34.87
SARS-CoV-2 #20	34.05	31.46
SARS-CoV-2 #21	36.10	35.33
SARS-CoV-2 #22	30.20	27.91
SARS-CoV-2 #23	36.00	33.74
SARS-CoV-2 #24	31.70	28.33
SARS-CoV-2 #25	35.70	33.10
SARS-CoV-2 #26	35.06	33.55
SARS-CoV-2 #27	35.00	32.45
SARS-CoV-2 #28	35.67	33.09
SARS-CoV-2 #29	34.30	32.67
SARS-CoV-2 #30	34.20	31.60

**Table 2:** Qualitative comparison of sample positivity and negativity between the two methods.

	In-house +	In-house -
Gold standard +	27	3
Gold standard -	0	30

**Table 3:** Results of linear regression for the in-house SARS-CoV-2 isolation methods.

	Estimated value
Slope (*m*)	-3.19
Intercept (*q*)	38.42
Correlation coefficient (*R*^2^)	0.963
*p* value	0.0005

**Table 4:** Reproducibility test for the in-house RNA isolation method.

	Sum of squares	Degrees of freedom (df)	Mean squares (MQ)	*F*-ratio	*p* value
Dilution 1	0.105	2	0.053	2.828	0.136
Dilution 2	0.089	2	0.045	1.928	0.226
Dilution 3	0.185	2	0.092	1.202	0.364

## Data Availability

Data sharing is not applicable to this article as no new data were created in this study.
